# Elevated FSP1 protects KRAS-mutated cells from ferroptosis during tumor initiation

**DOI:** 10.1038/s41418-022-01096-8

**Published:** 2022-11-29

**Authors:** Fabienne Müller, Jonathan K. M. Lim, Christina M. Bebber, Eric Seidel, Sofya Tishina, Alina Dahlhaus, Jenny Stroh, Julia Beck, Fatma Isil Yapici, Keiko Nakayama, Lucia Torres Fernández, Johannes Brägelmann, Gabriel Leprivier, Silvia von Karstedt

**Affiliations:** 1 University of Cologne, Faculty of Medicine and University Hospital Cologne, Department of Translational Genomics, Cologne, Germany; 2 CECAD Cluster of Excellence, University of Cologne, Cologne, Germany; 3 Heinrich Heine University, Medical Faculty and University Hospital Düsseldorf, Institute of Neuropathology, Düsseldorf, Germany; 4 Division of Cell Proliferation, ART, Graduate School of Medicine, Tohoku University, Sendai, Japan; 5 University of Cologne, Faculty of Medicine and University Hospital Cologne, Center for Molecular Medicine Cologne, Cologne, Germany; 6 Mildred Scheel School of Oncology Cologne, Faculty of Medicine and University Hospital Cologne, University of Cologne, Cologne, Germany

**Keywords:** Oncogenes, Cell biology

## Abstract

Oncogenic KRAS is the key driver oncogene for several of the most aggressive human cancers. One key feature of oncogenic KRAS expression is an early increase in cellular reactive oxygen species (ROS) which promotes cellular transformation if cells manage to escape cell death, mechanisms of which remain incompletely understood. Here, we identify that expression of oncogenic as compared to WT KRAS in isogenic cellular systems renders cells more resistant to ferroptosis, a recently described type of regulated necrosis. Mechanistically, we find that cells with mutant KRAS show a specific lack of ferroptosis-induced lipid peroxidation. Interestingly, KRAS-mutant cells upregulate expression of ferroptosis suppressor protein 1 (FSP1). Indeed, elevated levels of FSP1 in KRAS-mutant cells are responsible for mediating ferroptosis resistance and FSP1 is upregulated as a consequence of MAPK and NRF2 pathway activation downstream of KRAS. Strikingly, FSP1 activity promotes cellular transformation in soft agar and its overexpression is sufficient to promote spheroid growth in 3D in KRAS WT cells. Moreover, FSP1 expression and its activity in ferroptosis inhibition accelerates tumor onset of KRAS WT cells in the absence of oncogenic KRAS in vivo. Consequently, we find that pharmacological induction of ferroptosis in pancreatic organoids derived from the LsL-KRAS^G12D^ expressing mouse model is only effective in combination with FSP1 inhibition. Lastly, FSP1 is upregulated in non-small cell lung cancer (NSCLC), colorectal cancer (CRC) and pancreatic ductal adenocarcinoma (PDAC) as compared to the respective normal tissue of origin and correlates with NRF2 expression in PDAC patient datasets. Based on these data, we propose that KRAS-mutant cells must navigate a ferroptosis checkpoint by upregulating FSP1 during tumor establishment. Consequently, ferroptosis-inducing therapy should be combined with FSP1 inhibitors for efficient therapy of KRAS-mutant cancers.

## Introduction

The Ras proto-oncogenes (HRAS, NRAS and KRAS) are amongst the most frequently mutated genes across human cancers [1–3]. KRAS in particular is mutated in lung and pancreatic ductal adenocarcinoma (PDAC) as well as colorectal cancer. Point mutations within KRAS favor its active, GTP-bound state [2, 3]. Thereby, oncogenic forms of KRAS constitutively signal through the mitogen-activated protein (MAPK) pathway, PI3K and Rac1 signaling pathways endowing them with a variety of advantages including evasion of extrinsic apoptosis [4, 5]. It is established that KRAS-mediated cellular transformation requires the generation of reactive oxygen species (ROS) through elevated expression of NADPH oxidase 1 (Nox1) [6]. Yet, it is poorly understood how cells expressing mutated KRAS can mitigate the problem of ROS-induced cell death. We recently showed that cells expressing oncogenic KRAS upregulate the cystine/glutamate antiporter xCT (SLC7A11) upon hydrogen peroxide stimulation to promote cellular transformation [7]. Interestingly, xCT has been shown to protect cells from ferroptosis, an iron-dependent type of regulated necrosis executed by the accumulation of lipid ROS [8]. Cells are protected from ferroptosis by glutathione peroxidase 4 (GPX4) [9] which depends on glutathione (GSH) as an electron donor to reduce lipid hydroperoxides. GSH synthesis is coupled to the availability of intracellular cysteine which can be generated from cystine imported via xCT [10]. In addition, recent studies indicate that the Coenzyme Q10 (COQ10) oxidoreductase ferroptosis suppressor protein 1 (FSP1, formerly AIFM2) protects cells from ferroptosis through the generation of the lipid radical-trapping agent ubiquinol [11, 12]. Although ferroptosis has been suggested to represent a vulnerability in HRAS-mutant cells [13], GPX4 deletion in pancreatic intraepithelial neoplasia (PanINs) in genetically engineered mouse models driven by KRAS^G12D^ did not effectively kill PanINs [14] strongly suggesting KRAS^G12D^-driven PanINs to be protected from ferroptosis through unknown mechanisms.

Here, through the use of various independent isogenic cellular models expressing near-endogenous levels of oncogenic or wild type (WT) KRAS, we demonstrate that oncogenic forms of KRAS render cells more resistant to ferroptosis through NRF2-mediated FSP1 upregulation in vitro and during tumor initiation in vivo.

## Materials and methods

### Cell lines

The panel of “Rasless” mouse embryonic fibroblasts (MEFs) reconstituted with various oncogenic KRAS mutations (RPZ26216, RPZ25854, RPZ26198, RPZ26186, RPZ26425, RPZ26299, RPZ26295) was generated and kindly provided by the RAS Initiative at the Frederick National Laboratory for Cancer Research (FNLCR), US. Independently, Rasless MEFs were also obtained from M. Barbacid to generate KRAS point mutants from bulk sorting without deletion of the endogenous floxed KRAS allele. All MEFs were grown in Dulbecco’s modified Eagle’s (DMEM) + GlutaMAX™ medium (Gibco) with 4 µg/ml of blasticidin. NIH-3T3 cells expressing KRAS^G12V^ were generated and described previously [7]. NIH-3T3 stably expressing 4OHT-inducible HRAS^G12V^ and freshly isolated KRAS^G12D^-inducible mouse embryonic fibroblasts (MEFs) were cultured in DMEM (Gibco) supplied with 1% L-Glutamine (Sigma) and 1% Sodium Pyruvate (Sigma). Human pancreatic duct epithelial cells (HPDE) were kindly provided by A. Trauzold (University of Kiel) and cultured in 75% RPMI 1640/ medium in presence of 25% keratinocyte growth medium 2 (PromoCell). The human non-small cell lung cancer (NSCLC) cell line A549 and mouse Lewis lung carcinoma cell line (3LL) were kindly provided by Prof. Julian Downward and cultured in RPMI 1640 medium (Gibco). HEK-293T cells were cultured in DMEM medium (Gibco). All media were supplemented with 10% fetal calf serum (FCS) (Sigma Aldrich) and 1000 U/mL of both penicillin and streptomycin (Pen/Strep) (Sigma Aldrich). All cells were kept at 37 °C with 5% CO_2_ and tested for mycoplasma at regular intervals (mycoplasma barcodes, Eurofins Genomics).

### Reagents

Blasticidin (AppliChem GmbH), RSL3 (Selleckchem), ML210 (Tocris), ML162 (Caymann), Erastin (Biomol), Sulfasalazine (SAS) (MedChemExpress), Imidazole Ketone Erastin (IKE) (Sellekchem), Ferrostatin-1 (Sigma Aldrich), Liproxstatin-1 (Biozol), Necrostatin-1s (Abcam), zVAD (ENZO), iFSP1 (Cayman Chemicals), Tert-butylhydroquinone (TBHQ) (Sellekchem), AMG510 (MedChemExpress), ARS1620 (Chemgood), PD184352 (Sigma Aldrich), MK2206 (Sellekchem), DRAQ7 (Biolegend), BODIPY C11 (Invitrogen), H2DCFDA (Invitrogen), Dharmafect I (Dharmacon), Puromycin (Sigma), Doxycycline hydrochloride (Alfa Aesar), 4-hydroxy-tamoxifen (4OHT) (Sigma), Polybrene (Merck), CaCl2 (Sigma Aldrich), HBS (Sigma Aldrich), propidium iodide (Sigma).

### Antibodies

Ras (clone RAS10, #05-516; Millipore, 1:1000), GPX4 (Abcam, ab41787, 1:2,000), xCT (Abcam, ab37185, 1:2000), ß-Actin (Sigma, A1978, 1:10,000), GAPDH (Cell Signaling, #97166, 1:2000), FSP1 (previously described [11], kindly provided by M. Conrad, undiluted hybridoma supernatant), p44/42 MAPK (Erk1/2) (Cell Signaling, #9102 1:1000), phospho-p44/42 MAPK (Erk1/2) (Thr202/Tyr204) (Cell Signaling, #4370, 1:1000), HRP-conjugated secondary antibodies: goat-anti-mouse-HRP (Linaris GmBH, 20400-1 mg, 1:10,000), goat-anti-rabbit-HRP (Linaris GmBH, 20402-1 mg, 1:10,000), goat-anti-rat-HRP (Sigma, A9037-1 ml, 1:10,000).

### Plasmids

The packaging plasmids pCMV-VSV-G (#8454), pCMV-VSV-G (#8454), pMDLg/pRRE (#12251) were obtained from Addgene, P442-empty vector and P442-PLI-AIFM2-WT was kindly provided by J. P. Friedmann-Angeli, pLKO.1-empty vector and pLKO.1-shFSP1 were purchased from Merck (NM_153779/TRCN0000112139/pLKO.1). pCW-Puro-KRAS^G12D^ to generate doxycycline KRAS^G12D^-inducible HPDE cells was cloned from pCW (addgene #50661 [15]) by replacing the existing Cas9 gene by human KRAS^G12D^ cDNA.

### siRNA transfections

Two hundred microliters Opti-MEM (Gibco) and 1.5 µL Dharmafect Reagent I (Dharmacon) were mixed and incubated for 5–10 min at room temperature. 2.2 µL of siRNA (Stock 20 mM) (Dharmacon) were added to the mixture and incubated for another 30 min at room temperature. After incubation, 200 µL of the mixture was added to each well (6-well) plate and cells were plated on top. Knockdowns were incubated for 48–72 h, as indicated.

### Cell viability assays

For this assay, 5000 or 10,000 (for iFSP1 ± RSL3 viability assays) cells were plated per 96-well plate 24 h before treatment. Cell viability was determined by Cell Titer Blue assay according to the manufacturer’s instructions (Promega).

### Cell death assays (flow cytometry)

One day before treatment 45,000 cells (clonal and bulk-sorted MEFs), 55,000 cells (cells expressing either P442-empty vector or P442-PLI-AIFM2-WT) or 50,000 cells (3LLs) were plated in each well of a 24-well plate. For FSP1 siRNA knockdown, 40,000 cells were seeded 48 h before treatment. To determine cell death, adherent and detached cells were harvested and stained with propidium iodide (PI) (1 µg/ml) (Sigma Aldrich) in PBS (Thermo Fisher) supplemented with 2% FBS. PI-positive cells were quantified by flow cytometry using an LSR-FACS Fortessa (BD Bioscience) and FlowJo software (BD Bioscience).

### Live cell imaging (IncuCyte)

Five thousand, 7500 or 10,000 cells per 96-well plate, 55,000 cells per 24-well plate or 300,000 cells per 6-well plate were seeded 24 h in advance, respectively. For KEAP1 siRNA knockdown, 20,000 cells were seeded in a 24-well plate on top of the transfection mix and incubated for 48 h followed by treatments for 24 h. Upon treatment, (Ferrostatin-1 [1 or 5 µM], RSL3 [0.1 µM or 1 µM], iFSP1 [10 µM], Erastin [0.37 µM], Sulfasalazine (SAS) [0.17 mM], Imidazole Ketone Erastin (IKE) [1.11 µM], ML210 [0.37 µM], ML162 [1.11 µM], TBHQ [25 nM]) cells were imaged using the 10× objective within the IncuCyte live cell imager (Sartorius). For dead cell quantification, 100 nM DRAQ7 (Thermofisher) were added to each well. For lipid ROS determination, cells were stained with 5 µM BODIPY C11. Cells were imaged for indicated timepoints every 2 h. Analysis for confluence, DRAQ7-positive (dead) or BODIPY C11-positive cells was performed using the Software IncuCyte 2021A (Sartorius).

### Quantitative PCR

For KRAS^WT^ and KRAS^G12D^ comparison, 300,000 cells were seeded per well in a 6-well plate and RNA was extracted 24 h later. For MEK and AKT inhibition treatment experiments 200,000 cells were seeded in a 6-well plate one day in advance followed by treatments for 48 h. For TBHQ treatment experiments, 150,000 of KRAS^WT^ cells were seeded in a 6-well plate a day in advance followed by treatment for 24 h. For KEAP1 knockdowns, 200,000 of KRAS^WT^ cells were seeded in a 6-well plate on top of the transfection mix and incubated for 72 h. For LsL-KRAS^G12D^-inducible MEFs, 200,000 cells were seeded in 1 µg/ml 4OHT in a 6-well plate and incubated for 4 or 5 days. For doxycycline-inducible KRAS^G12D^ HPDE cells, 450,000 cells were seeded in 0.5 µg/mL doxycycline in a 6-well plate and incubated for 72 h. For 4OHT-inducible HRAS^G12V^ NIH-3T3 cell, 35,000 cells were seeded for 72 h in a 6-well plate and 100 nM 4OHT was added and incubated for another 48 h.

For total RNA isolation, the NucleoSpin RNA kit (740955.5, Macherey-Nagel) was used according to the manufacturer’s instructions. Next, isolated RNA was reverse transcribed into cDNA using the LunaScript RT SuperMix Kit (E3010L, NEB). For quantitative PCR, 5 µl of Power SYBR GREEN PCR Master Mix (4368702, Thermo Fisher) was mixed with 2 µl of nuclease-free water (NEB), 1 µl (10 µM) of primer mix (forward and reverse primers) (see Supplementary Table 1 for primers used) and 2 µl of cDNA (5 µg/µl). Real-time qPCR was performed in triplicate or in quadruplicate on the Quant Studio5 qRT PCR cycler and results were normalized to the expression of the house-keeping gene indicated. Actin, Rplp0, Rpl13a or 18S were used as house-keeping gene controls as indicated.

### Lipid ROS quantification (flow cytometry)

Thirty-five thousand cells per well were plated in a 24-well plate 24 h before treatment. Lipid ROS levels were quantified by BODIPY C11 (Invitrogen) staining. To this end, cells were stained using 5 µM BODIPY C11 during the last 30 min of treatment incubation. Mean fluorescence intensity (MFI) was determined by flow cytometry using an LSR-FACS Fortessa (BD Bioscience) and FlowJo software (BD Bioscience). Flow cytometry data were collected from at least 5000 cells.

### General ROS quantification (flow cytometry)

Fifty-five thousand cells per well were plated in a 24-well plate 24 h before treatment. Cells were incubated with 20 µM H2DCFDF (Invitrogen) per well to stain cellular ROS. Mean fluorescence intensity (MFI) was determined by flow cytometry using an LSR-FACS Fortessa (BD Bioscience) and FlowJo software (BD Bioscience). Flow cytometry data were collected from at least 5000 cells.

### NADPH Assay

Twenty thousand cells per 96-well plate were seeded in advance. NADP/NADPH was determined by NADP/NADPH-Glo™ Detection Reagent assay according to the manufacturer’s instructions (Promega).

### Lipidomics to determine oxidized lipids and levels of total phospholipids

Mass spectrometry experiments to determine total phospholipids and oxidized lipids were performed as described previously [16]. In brief, levels of oxidized phosphatidylcholine (PC) and phosphatidylethanolamine (PE) species were determined by Liquid Chromatography coupled to Electrospray Ionization Tandem Mass Spectrometry (LC-ESI-MS/MS). Oxidized PC and PE species were quantified by normalizing their peak areas to those of the internal standards. Glycerophospholipids (PC and PE, including ether-linked species) in cells were analyzed by Nano-Electrospray Ionization Tandem Mass Spectrometry (Nano-ESI-MS/MS) with direct infusion of the lipid extract (Shotgun Lipidomics). The protein content of the homogenate was routinely determined using bicinchoninic acid. Endogenous glycerophospholipids were quantified by referring their peak areas to those of the internal standards. The calculated glycerophospholipid amounts were normalized to the protein content of the cell homogenate.

### Western blotting

For KRAS^WT^ and KRAS^G12D^ 300,000 cells were seeded one day in advance in a 6-well plate before cells were treated for another 5 h with or without RSL3. For FSP1 knockdowns, 75,000 cells were seeded in a 6-well plate on top of the transfection mix and incubated for 72 h. For KRAS^WT^ and KRAS^G12D^ cells expressing either empty Vector or FSP1-WT 300,000 cells were seeded in a 6-well plate for 24 h. For LsL-KRAS^G12D^-inducible MEFs, cells were incubated for 72 h with or without 1 µg/ml 4OHT before 250,000 cells were seeded into a 6-well plate for indicted timepoints. For MEKi and AKTi treatment experiments 150,000 cells were seeded one day in advance in a 6-well plate before cells were treated for 72 h. Cell lysates were prepared in lysis buffer (30 mM Tris-HCl [pH 7.4], 120 mM NaCl, 2 mM EDTA, 2 mM KCl, 1% Triton X-100, 1× COMPLETE protease-inhibitor and phosphatase- inhibitor cocktail). Lysate concentrations were adjusted to equal protein concentrations using the bicinchoninic acid (BCA) protein assay (Biorad). Equal amounts of protein were mixed with a final concentration of 1× reducing sample buffer (Invitrogen) and 200 mM DTT (VWR). Samples were heated to 95 °C for 10 min, separated via gel electrophoresis and transferred to nitrocellulose membranes (Biorad). Membranes were blocked in PBS with 0.1% Tween 20 (PBST) (VWR) with 5% (w/v) dried milk powder (AppliChem) for at least 30 min and incubated with primary antibodies over night. After washing with PBST, membranes were incubated with horse radish peroxidase (HRP)-coupled secondary antibodies (Biotium) diluted 1:10,000 for at least 1 h at room temperature. After another washing step, bound antibodies were detected using chemiluminescent Amersham ECL Prime Western Blotting Detection Reagent (Cytiva) or SuperSignal™ West Femto Maximum Sensitivity Substrate (Thermo Fisher). X-ray films CL-XPosure™ (Thermo Fisher) or the FUSION Solo S system and software (Vilber) were used to develop the membranes.

### RNA sequencing

For RNA sequencing, 70,000 cells per well (6-well plate) of either KRAS^WT^ or KRAS^G12D^ were seeded 24 h in advance. The next day, cells were washed with PBS and RNA was isolated using the NucleoSpin RNA kit (740955.5, Macherey-Nagel) according to the manufacturer’s instructions. cDNA libraries amplified from the 3′ UTR were generated from total RNA using the Lexogen QuantSeq kit (Lexogen, Austria) according to the standard protocol and sequenced with a 50-bp single-end protocol on Illumina HiSeq4000 sequencer (Illumina, USA). The raw-sequencing data was aligned to the respective mouse reference genomes and quantified prior to differential expression analyses. Raw FPKM values of each transcript were transformed by log_2_ (FPKM + 0.01). Data processing and statistical analyses were performed using Microsoft Excel (Microsoft, USA) and Instant Clue software [17] which performed a hierarchical clustering to classify the experiments and generate a heatmap for the visualization of different RNA expression.

### Generation of FSP1-overexpressing cells

To generate stable cells overexpressing FSP1, viral particles were produced in HEK-293T cells. HEK-293T cells were plated in a 6-well plate the day before transfection. For 6 × 6 wells of a 6-well plate 5 µg of each packaging plasmid and 10 µg of transfer vector plasmid were mixed together. Fifty microliters of 250 mM CaCl_2_ and 444 µl H_2_O were added to the plasmid mixture and mixed well by pipetting. For the formation of calcium-phosphate-DNA co-precipitate, the plasmid transfection mix (~500 µl) was carefully dropped into 500 µl of 2× HBS buffer under constant vortexing. The precipitate was incubated for 20 min at room temperature and added dropwise into freshly replaced media without Pen/Strep. After 6–8 h of transfection medium was aspirated, and fresh normal medium was added. The following two days, virus-containing supernatant was harvested and filtered with 0.45 μm sterile syringe filter. Fresh medium was always added again on the cells. Virus harvest was centrifuged, and supernatant was collected and stored at −80 °C. For transduction of the KRAS^WT^ and KRAS^G12D^ cells viral supernatant was added to wells containing cells with 6 µg/ml polybrene and centrifuged for 45 min at 2500 rpm at 30 °C. Cells were incubated afterwards at 37 °C and 5% CO_2_ until they were confluent and selected for positively-transfected cells using Puromycin (KRAS^WT^ 1 μg/ml and KRAS^G12D^ 2.5 μg/ml) for 7 days.

### Generation of stable FSP1 knockdown cells

For stable transduction of FSP1 knockdown cells, lentiviral supernatant with an shFSP1 transfer plasmid was produced as described above and KRAS^G12D^ cells were transduced with the virus. After selection with Puromycin (2.5 μg/ml) for 7 days, knockdown was validated using qPCR.

### Generation of LsL-KRAS^G12D^-inducible MEFs

Mouse embryonic fibroblasts (MEFs) were generated from E13.5 mouse embryos by standard Jacks lab procedure (https://jacks-lab.mit.edu/protocols/making_mefs). MEFs with positive genotypes for 4-hydroxytamoxifen (4OHT)-inducible Cre (primer: #1 GCG GTC TGG CAG TAA AAA CTA TC, #2 GTG AAA CAG CAT TGC TGT CAC TT) and LsL-KRas^G12D^ (primer: #1 gtc ttt ccc cag cac agt gc, #2 ctc ttg cct acg cca cca gct c; #3 agc tag cca cca tgg ctt gag taa gtc tgc a) were used for the experiments. To obtain Cre expression cells were treated with 1 µg/ml 4OHT.

### Generation of HRAS^G12V^-inducible NIH-3T3

NIH-3T3 stably expressing 4OHT-inducible HRAS^G12V^generated as previously described [18]. Activation of Ras was induced by exposure of cells to 100 nM 4OHT, with 4OHT-supplemented medium being refreshed every two days.

### Generation of KRAS^G12D^- inducible human pancreatic duct epithelial cells (HPDE)

Lentiviral particles were produced in HEK-293T cells transfected with packaging plasmids pCMV-VSV-6, pMDLg and pRSV-Rev as well as 10 µg pCW-Puro-KRAS^G12D^ (a newly cloned pCW (addgene #50661 [15]) backbone replacing the existing Cas9 by human KRAS^G12D^ cDNA) using a 1:1 mixture of 2× HBS and 250 mM CaCl2 in DMEM F12 medium with 10% FCS, 1% Pen/Strep and 1% Glutamine. After overnight incubation, medium was replaced for HPDE medium (see above), and medium containing viral particles was subsequently harvested after 48 h. HPDE cells were infected in a 6-well plate at 30% confluency by replacing the medium by viral supernatant after adding polybrene at a final concentration of 8 µg/mL. After 3 days, medium was replaced by selection medium containing 0.5 µg/mL puromycin. For KRAS^G12D^ induction, HPDE cells were treated with 0.5 µg/ml doxycycline for 72 h.

### Spheroid assays

To generate spheroid cultures, 5000 cells per 96-well were plated in a 96 ultra-low attachment multi-well plate (Corning) in 100 µl media containing 4 % Matrigel (Corning). After 24 h 2× iFSP1 [10 µM or 20 µM], 2× Fer1 [2.5 µM] or 2× Liproxstatin-1 [0.3 µM] treatment in 100 µl media was added to the cells and incubated for 9 (A549) or 14 days (MEFs). Pictures of the spheroids were taken with the BZ-X800E microscope (Keyence). Spheroid assay and organoid assay colony area and brightness were analyzed using the BZ-H4M/Measurement Application Software (Keyence).

### Soft agar colony formation assays

Cells were plated in 6-well plates at 8000 cells per well. Equal volumes of culturing medium and agarose were used such that the final concentrations were DMEM, 10% bovine serum (for 3T3 KRAS) or RPMI, 10% fetal bovine serum (for A549) and 0.25% agarose for the top layer or 0.4% agarose for the bottom layer, respectively. Where indicated, DMSO, iFSP1 (10 or 20 µM) or Fer1 [5 µM] was added to the top agar layer. Cells were fed twice a week with 1 mL of corresponding DMSO or iFSP1 treated medium onto the top layer. Colonies were allowed to form over the course of 18 to 30 days, following which they were imaged and quantified using ImageJ.

### Tumor xenograft studies

Mice were maintained on a 12-h light/dark cycle with water and food *ad libitum* throughout the duration of the project. Mouse embryonic fibroblast (MEF) cell lines (5 × 10^5^ cells either KRAS^WT^ e.V., KRAS^WT^ FSP1-WT, KRAS^G12D^ e.V. or KRAS^G12D^ shFSP1) were injected in 200 µl PBS into both flanks of 8–10 weeks old male NMRI-Foxn1 nu/nu mice (Janvier). Mice were not randomized. A group size of at least 10 tumors per condition was assumed to achieve significantly different results (*p* = 0.05) with a power of 80%. For that, cells were harvested from plates using trypsin and washed five times with PBS to remove residual FCS. Mice injected with KRAS^WT^ e.V. or KRAS^WT^ FSP1-WT were assigned to either vehicle or Liproxstatin-1 treatment groups once tumors reached a minimum size of 2.5 × 2.5 mm. For two consecutive weeks, mice were injected 5 × per week either with vehicle (PBS with 1% DMSO) or Liproxstatin-1 (10 mg/kg). Tumor size was tracked by caliper measurements and volume was calculated as (length × width × width)/2. People performing tumor measurements and calculating tumor volume were blinded to the group allocation. Mice were sacrificed at the end of the treatment and fresh-frozen tumor tissue was used for further analysis.

### Protein extraction from fresh-frozen tumors

For protein isolation, 20–30 mg of fresh-frozen tumor tissue were mixed in a peqlab vial with the adequate number of ceramic beads and 500 µl IP-lysis buffer (30 mM Tris-HCl, 120 mM NaCl, 2 mM EDTA, 2 mM KCl, 1% Triton-X-100, pH 7.4, Protease and Phosphatase inhibitor (Roche)). For lysis, samples were homogenized for 2 × 30 s using the Precellys 24-dual homogenisator (Peqlab). Samples were centrifuged at 14,000 RPM for 20 min at 4 °C and then further used for western blotting.

### Isolation and treatment of murine pancreatic organoids

The pancreas was isolated from PDX1-Cre KRAS ^G12D^ mice, washed with cold mouse wash medium (DMEM high glucose + Pen/Strep + 1% FCS) and cut into 1–2 mm pieces using scalpels. Pancreatic pieces were transferred into 50 ml falcons containing 10 ml of mouse digestion medium (200 ml Mouse wash medium + 25 mg Collagenase P; Sigma-Aldrich #C9407 + 25 mg Dispase II; Thermo Fisher #17105041) and shaken at 130 rpm at 37 °C for 20 min. The supernatant was transferred to a Petri dish containing 10 ml of mouse washing medium to obtain the first wash fraction. Ten milliliters of mouse digestion medium were added to the remaining pancreatic pieces. Cycles of shaking at 130 rpm at 37 °C for 10 min were repeated until wash fractions with mainly pancreatic ducts and almost no acinar cells were observed. Wash fractions enriched with ducts were combined and spun down at 1200 rpm for 5 min. The obtained duct pellet was resuspended in 200 µl of ice-cold Matrigel (Growth Factor Reduced, Phenol Red Free; Corning #356231) and a 30 µl dome was seeded into the middle of a well in a prewarmed 24-well plate. Ducts were consecutively diluted in Matrigel to obtain a cellular density with the most favorable conditions for organoids growth. The plate containing domes was placed into the cell culture incubator for 10–15 min for the Matrigel to set before 500 µl of PancreaCult™ Organoid Growth Medium (Mouse) (Stemcell #06040) was added. The organoids were maintained in cell culture with splitting once a week and twice-weekly medium change. For organoids treatments, single cells from 5 days old PDX1-Cre KRAS ^G12D^ organoids were isolated according to Boj et al. [19] and Huch et al. [20] and seeded at 200 cells in 100 µl medium/well in a 96-well plate covered in Matrigel:DPBS (1:1). Organoids from single cells were left to grow for 6 days, then treated with 50 µl of PancreaCult medium containing the indicated treatments for 2 days before microscopic pictures were taken using the BZ-X800E microscope (Keyence).

### Analysis software and bioinformatic analysis

Heatmaps visualizing ferroptosis and KEGG pathway component expression were generated using Instant Clue software [17]. FACS data were analyzed and quantified using the FlowJo 10.4.2 software. Cell Titer Blue viability assays and qPCR results were analyzed using Excel. Lipidomics measurements were analyzed by MultiQuant 3.0.2 software (SCIEX). IncuCyte experiments were analyzed by using the Software IncuCyte 2021A. Soft Agar colonies were imaged and quantified using ImageJ. Spheroid assay and organoid assay colony area and brightness were analyzed using the BZ-H4M/Measurement Application Software (Keyence). Figures were assembled and data plotted and analyzed using GraphPad Prism 7 for Mac OS X.

### Quantification, statistical analysis and reproducibility

GraphPad Prism 7 software (GraphPad Software Inc.) was used for Mac OS X to execute statistical analysis. For comparison between two conditions two-tailed *t*-tests were performed and for comparison between multiple samples two-way ANOVA and Tukey’s post test for multiple comparisons were used. All data are presented as mean ± SEM of at least three independent biological replicates. From at least three independent experiments all means are calculated and plotted. Biological replicates gave comparable results, and no technical or biological replicates were excluded. In the respective figure legends statistical tests are declared. The following *p* value cut-offs were used for all tests: ****p < 0.0001, ***p < 0.001, **p < 0.01, *p < 0.05, ^ns^*p* > 0.05. Representative western blots are shown.

## Results

### Endogenous-level expression of oncogenic KRAS protects from ferroptosis

**Fig. 1:**
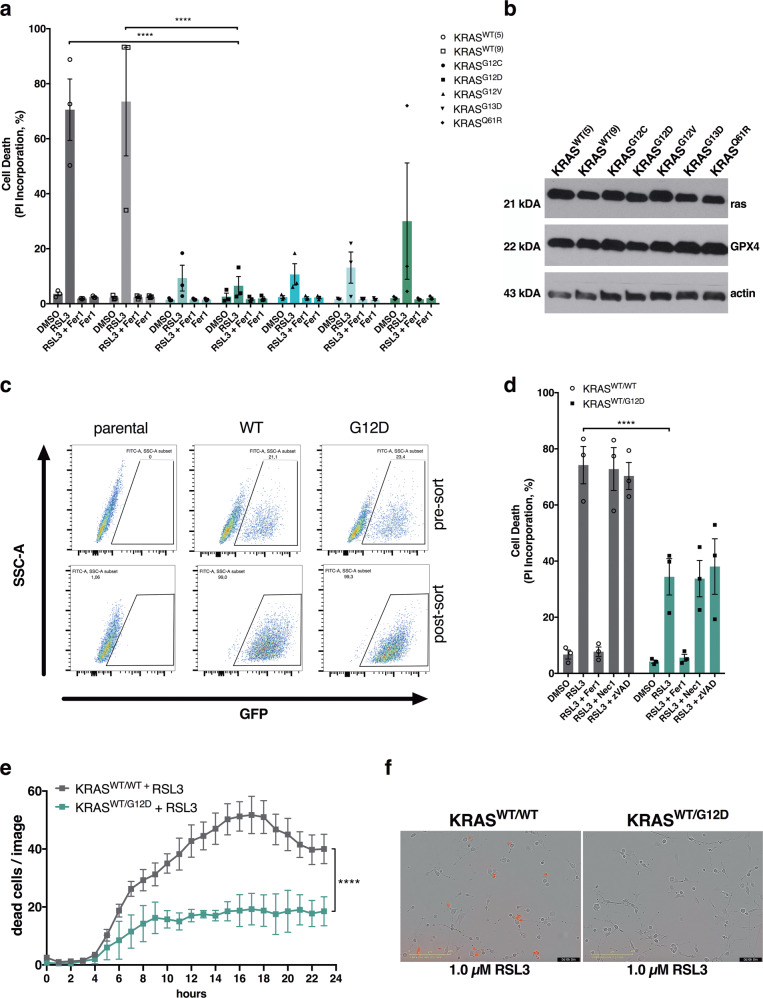
Expression of oncogenic KRAS renders cells resistant to ferroptotic cell death. **a** Rasless MEFs expressing the indicated variants of either KRAS WT or mutants were treated either with DMSO, RSL3 [100 nM] alone or in combination with Ferrostatin-1 (Fer-1) [5 µM] for 24 h. Cell death was determined by propidium iodide (PI) uptake and flow cytometry. 0 % PI-Incorporation is gated to control untreated. **b** Protein extracts were obtained from cells as in (**a**) and expression of the indicated proteins was detected by Western blotting. **c** Parental Rasless MEFs were infected with viral supernatants containing the indicated stable expression plasmids. FACS dot plots before and after sorting for GFP + cells are shown. **d** Bulk-sorted cells as in (**c**) were treated as in (**a**) but including Necrostatin-1s (Nec1) [10 µM] and zVAD [20 µM]. 0 % PI-Incorporation is gated to untreated control. **e** Cells as in (**c**) were treated with RSL3 [1 µM] and DRAQ7 [100 nM] was added to all wells to visualize dead cells. Images were acquired at ×10 magnification every 2 h using the IncuCyte S3 bioimaging platform. **f** Representative phase contrast overlays are shown from cells treated as in (**e**). Data are means ± SEM of three independent experiments in each individual cell line or representative images were applicable. Two-way ANOVA + Tukey’s multiple comparison test (**a, d**), two-tailed *t*-test at end timepoint (**e**), ****p < 0.0001. Uncropped blots are provided as Original Data file.

In order to interrogate the influence of oncogenic KRAS expression in genetically defined isogenic cellular systems, we made use of N- and HRAS-deficient mouse embryonic fibroblasts (MEFs) containing a LoxP-flanked KRAS gene as well as tamoxifen-inducible Cre recombinase. After induction of Cre recombinase- which renders these cells “Rasless” [21]- cells were reconstituted with comparable expression levels of either wild type KRAS 4B or commonly mutated forms of KRAS 4B (hereafter referred to as KRAS; cell line panel available from the Ras initiative at the NIH national cancer institute, US). Strikingly, when treating this cell line panel with the GPX4 small molecule inhibitor RSL3, both WT KRAS-expressing clones were killed within 24 h of treatment whilst all cells expressing oncogenic variants of KRAS were more resistant (Fig. 1a). Moreover, co-treatment with the ferroptosis-selective antioxidant ferrostatin-1 (Fer-1) [8], blocked cell death induced in KRAS WT cells confirming the induction of ferroptosis (Fig. 1a). Importantly, this phenotype was not caused by varying levels in KRAS or expression of GPX4, as all cell lines expressed comparable levels of both proteins (Fig. 1b). Moreover, treatment with other class II ferroptosis-inducing compounds (FINs) which directly inhibit GPX4 equally led to a more drastic loss of viability in KRAS WT cells as compared to oncogenic KRAS while for class I FINs no significant difference could be observed (Supplementary Fig. 1a). In addition, loss of cell confluence induced by RSL3 as well as cell death was consistently less pronounced over time also in live cell imaging kinetic experiments in a representative KRAS^G12D^-mutated MEF line (Supplementary Fig. 1b, c). As these MEFs are generated from single cellular clones, we independently generated Rasless MEFs from bulk-sorted populations to exclude clonal effects. These express near endogenous levels of FLAG-tagged KRAS behind GFP and an internal ribosomal entry site (IRES) and were enriched via bulk sorting of GFP^+^ cells (Fig. 1c). Importantly, expression of oncogenic KRAS^G12D^ but not KRAS WT equally rendered cells more resistant to ferroptotic cell death in bulk-sorted MEFs (Fig. 1d). Of note, ferrostatin-1 but not the caspase inhibitor zVAD or the RIPK1 inhibitor nec-1s could block cell death induced by RSL3 indicating ferroptotic cell death. Moreover, WT KRAS-expressing cells also died more rapidly upon RSL3 treatment than KRAS-mutant cells (Fig. 1e, f). In order to determine whether direct signaling from oncogenic KRAS is responsible for increased ferroptosis resistance we made use of the fact that effective small molecule inhibitors against KRAS^G12C^ have recently been developed [22–25]. Indeed, treating either KRAS^G12C^-expressing MEFs or the established Lewis lung carcinoma cell line (3LL) - in which NRAS was knocked out (deltaNRAS86) leaving them only with an activating point mutation in KRAS^G12C^ [26] - with the KRAS^G12C^ inhibitor AMG510 sensitized these cells to ferroptosis (Supplementary Fig. 1d, e). Taken together, we find that expression of near-endogenous levels of oncogenic KRAS renders cells more resistant to ferroptotic cell death in various isogenic cellular and experimental setting.

### KRAS-mutated cells are protected from ferroptosis-induced lipid peroxidation

**Fig. 2:**
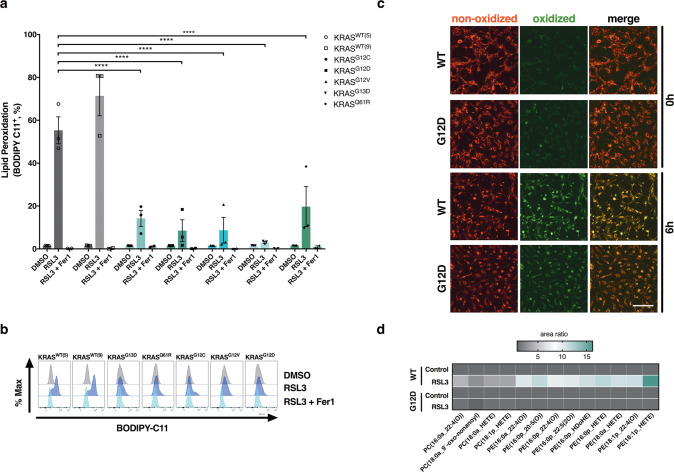
Expression of oncogenic KRAS protects cells from ferroptosis-associated lipid ROS accumulation. **a** Rasless MEFs expressing the indicated variant of KRAS were treated either with DMSO, RSL3 [100 nM] alone or in combination with Ferrostatin-1 (Fer-1) [5 µM] for 5 h and stained for lipid ROS accumulation using BODIPY C11. Cells were analyzed by flow cytometry. Negative gates were placed based on DMSO controls. **b** Representative histograms from cells in (**a**) are shown. **c** Rasless MEFs expressing WT or KRAS^G12D^ were treated with RSL3 [100 nM] and stained using BODIPY C11. Images were acquired every 2 h using the IncuCyte S3 bioimaging platform. Representative red, green and overlay fluorescent images (100×) are shown at 0 h and 6 h after treatment. **d** Heatmap showing the representation of mono-oxidized phospholipid species (PE phosphatidylethanolamine; PC phosphatidylcholine) in KRAS WT as compared to KRAS^G12D^-expressing cells treated with either DMSO or RSL3 [100 nM] for 5 h and then subjected to lipidomics. Samples for each condition (*n* = 5) were averaged and normalized to the cell number (2.5 × 10^6^). Each lipid species was normalized to levels detected in the respective DMSO control. Data are means ± SEM of three independent experiments in each individual cell line or representative images or histograms were applicable. Two-way ANOVA + Tukey’s multiple comparison test (**a**), ****p < 0.0001.

A major hallmark of ferroptosis is a lipid ROS-dependent lipid peroxidation chain reaction [27] which oxidizes phosphatidylethanolamine (PE) and phosphatidylcholine (PC) species containing arachidonic (AA) and adrenic acid (AdA) [28, 29]. Since we observed that cells expressing various forms of mutated KRAS were more resistant to ferroptosis than KRAS WT cells, we next determined the extent of lipid ROS accumulation. Indeed, both WT KRAS clones readily accumulated lipid ROS 5 h after stimulation with RSL3 while all KRAS mutants tested did not show lipid ROS accumulation at this time (Fig. 2a, b). Bulk-sorted MEFs expressing flag-tagged mutant KRAS^G12D^ but not WT KRAS, similarly presented with decreased lipid ROS accumulation upon inhibition of GPX4 (Supplementary Fig. 2a). Moreover, KRAS-mutated cells also showed a decrease in the accumulation of oxidized BODIPY C11 indicative of the presence of lipid ROS in time-lapse imaging (Fig. 2c). These data suggested that oncogenic KRAS limits the propagation of lipid ROS and thereby acute lipid peroxidation upon induction of ferroptosis. As the extent of lipid peroxidation during ferroptosis is coupled to cellular amounts of AA-containing polyunsaturated fatty acid (PUFA) PE and PC species including ether-linked PUFAs [30], we first measured basal levels of diacylglycerol (DAG) and ether-linked PE and PC PUFAs in KRAS WT as compared to mutant cells using mass spectrometry. While levels of most PUFA species were comparable, a few PC species were elevated in KRAS WT cells (Supplementary Fig. 2b–e). Next, we determined the levels of phospholipid oxidation upon ferroptosis induction in Rasless MEFs expressing either KRAS WT or KRAS^G12D^ by mass spectrometry. Although we observed elevated basal lipid ROS in KRAS-mutated cells (Supplementary Fig. 2f) -likely as a result of elevated basal levels of total ROS due to oncogene expression (Supplementary Fig. 2g) - specific induction of lipid peroxidation upon GPX4 inhibition was absent in KRAS-mutated cells (Fig. 2d). Interestingly, total ROS was nevertheless readily induced in KRAS-mutated cells by GPX4 inhibition (Supplementary Fig. 2g) suggesting the protection from ROS to be specific towards lipid ROS. Together, these data establish that mutant KRAS endows cells with superior capacity to protect cells from a ferroptosis-specific increase in lipid peroxidation.

### Elevated levels of FSP1 protect KRAS-mutated cells from ferroptosis

**Fig. 3:**
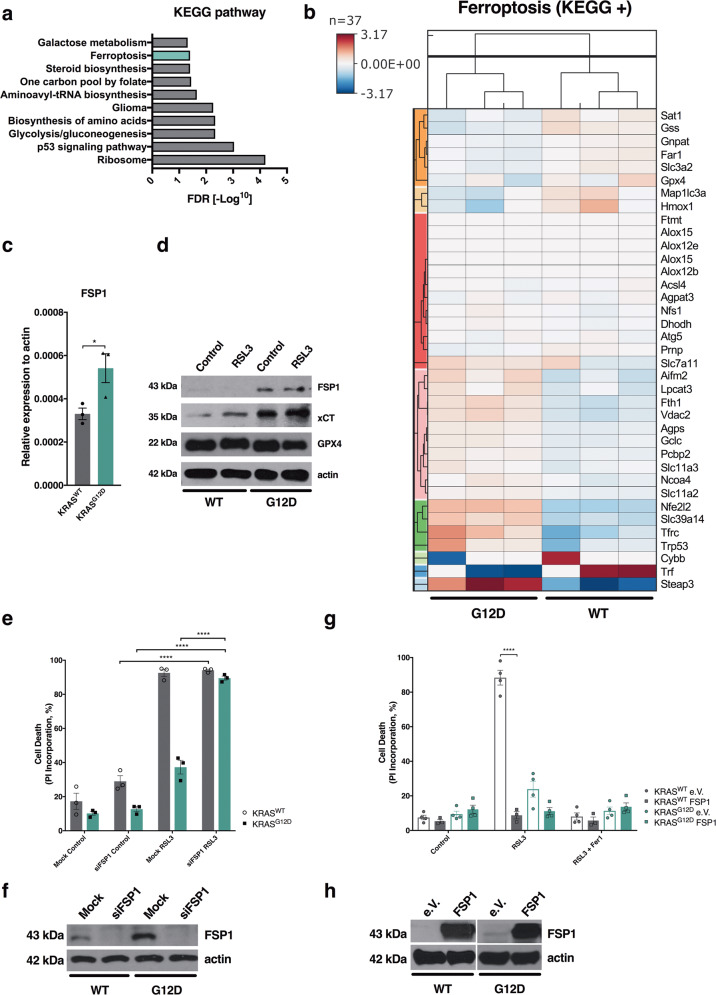
FSP1 is upregulated in KRAS^G12D^-expressing cells and mediates ferroptosis resistance. **a** KRAS WT or KRAS^G12D^-expressing cells were subjected to RNA-sequencing. False discovery rate (FDR) [−Log^10^] is shown for KEGG pathways significantly enriched within the top 1000 genes upregulated in KRAS^G12D^ cells. **b** Hierarchical clustering of fold change (FPKM + 0.01) of ferroptosis KEGG + genes in KRAS WT and KRAS^G12D^-expressing cells. **c** Levels of FSP1 mRNA were quantified by qPCR in Rasless MEFs expressing WT or KRAS^G12D^ **d** Indicated cells were treated with RSL3 [100 nM] for 5 h and subjected to Western blotting. **e, f** The indicated cells were subjected to FSP1 or control knockdowns for 48 h and subsequently treated with RSL3 [100 nM] for 24 h. Cell death was determined by flow cytometry and propidium iodide (PI) incorporation. 0 % PI-Incorporation is gated to untreated control. Western blots of representative control lysates are shown. **g, h** WT and KRAS^G12D^ cells stably overexpressing FSP1 were generated and cells were treated with RSL3 [100 nM] alone or in combination with Ferrostatin-1 (Fer-1) [5 µM] for 24 h. Cell death was determined by propidium iodide (PI) uptake and flow cytometry. 0 % PI-Incorporation is gated to control untreated. Western blots of representative control lysates are shown. Data are means ± SEM of three independent experiments in each individual cell line or representative images or histograms were applicable. Two-tailed t-test (**c**), Two-way ANOVA + Tukey’s multiple comparison test (**e, g**), ****p < 0.0001, *p < 0.05. Uncropped blots are provided as Original Data file.

In order to determine the mechanism by which KRAS-mutated cells might buffer acute lipid peroxidation, we performed comparative 3′ RNA sequencing of KRAS WT and KRAS^G12D^-mutated MEFs. Interestingly, when analyzing the top 1000 upregulated genes in KRAS-mutated cells for functional association networks using STRING, we obtained a significant enrichment of the ferroptosis pathway along with several other metabolic pathways (Fig. 3a). As the list of genes annotated in the KEGG ferroptosis pathway does not contain more recently discovered regulators of ferroptosis, we manually extended this list (KEGG+) and analyzed expression of these genes in our comparative dataset. Strikingly, AIFM2 mRNA, recently renamed as ferroptosis suppressor protein 1 (FSP1) due to its ferroptosis protective activity [11, 12], was upregulated in KRAS-mutated cells within a cluster of genes (Fig. 3b). Within several ferroptosis regulatory genes of the dataset, FSP1 was upregulated significantly (Supplementary Fig. 3a). Moreover, FSP1 mRNA upregulation in KRAS-mutated cells could also be confirmed by quantitative real-time PCR (qPCR) (Fig. 3c). Importantly, FSP1 was basally upregulated also on protein level in KRAS-mutated cells and, unlike xCT, a recently identified target gene further upregulated upon H2O2 [7], was not further increased upon stimulation with RSL3 (Fig. 3d). As FSP1 has been shown to render cells more resistant to ferroptosis, we next tested whether its elevated expression in KRAS-mutated cells was responsible for mediating increased ferroptosis resistance of KRAS-mutated cells. Indeed, FSP1 suppression was sufficient to sensitize KRAS-mutated cells to ferroptosis (Fig. 3e, f). Vice versa, overexpression of FSP1 was sufficient to render KRAS WT cells as resistant to ferroptosis as KRAS-mutated control cells (Fig. 3g, h). As FSP1 is an NADH ubiquinone oxidoreductase, FSP1 activity requires NADPH as an electron source [11, 12]. Therefore, we also tested whether basal levels of the FSP1 cofactor NADPH would differ in KRAS-mutated as compared to WT cells and thereby contribute to differential activity, yet this was not the case (Supplementary Fig. 3b). To next determine whether FSP1 activity may also protect KRAS-mutated cells from ferroptosis, we employed a recently developed small molecule inhibitor against FSP1 [11]. Strikingly, co-incubation with this inhibitor (iFSP1) reverted ferroptosis resistance endowed by oncogenic KRAS expression (Supplementary Fig. 3c, d). Of note, iFSP1 also slightly sensitized KRAS WT cells, yet due to the fact WT cells were already very sensitive, the relative sensitization observed was much stronger for KRAS-mutated cells. Thus, KRAS-mutated cells display increased ferroptosis resistance due to elevated levels of FSP1.

### Oncogenic KRAS upregulates FSP1 via NRF2 and the MAPK pathway

**Fig. 4:**
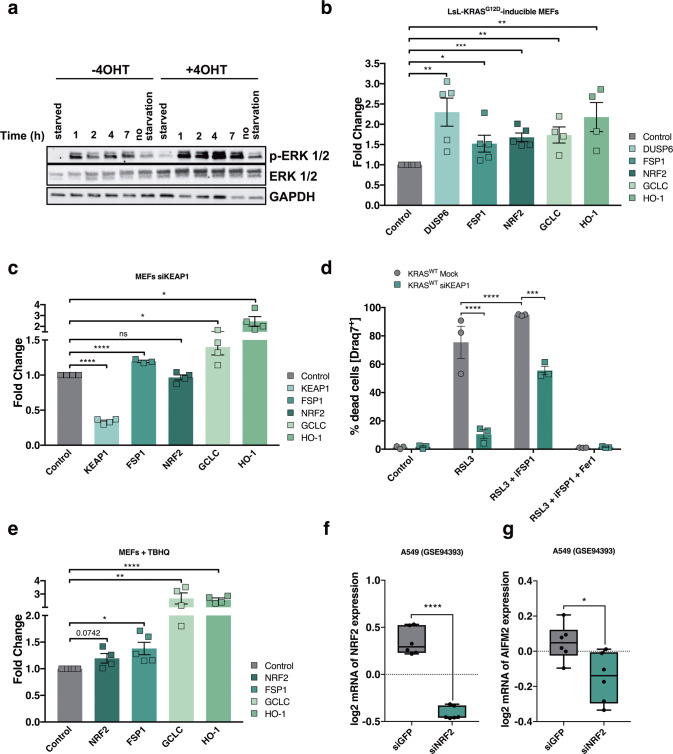
FSP1 is upregulated in KRAS^G12D^-expressing cells in an NRF2-dependent manner. **a** LsL-KRAS^G12D^-inducible MEFs were treated for 72 h with or without tamoxifen (4OHT) [1 µg/ml] in 2% FCS before cells were starved overnight in 0.1% FCS and then refed with 2% FCS for the indicated timepoints. Cells were lyzed and subjected to protein analysis by Western blotting. **b** Levels of DUSP6, FSP1, NRF2, GCLC and HO-1 cDNA were quantified by qPCR in LsL-KRAS^G12D^-inducible MEFs after 96 h or 120 h of tamoxifen (4OHT) treatment. Fold change relative to controls is shown. Means from MEF lines from 4–5 different embryos are shown. **c** Levels of KEAP1, FSP1, NRF2, GCLC and HO-1 cDNA were quantified by qPCR in Rasless MEFs expressing KRAS WT ± KEAP1 knockdowns for 72 h. Fold change relative to controls is shown. **d** siKEAP1 KRAS WT cells were treated after 48 h knockdown with DMSO, RSL3 [100 nM], ±iFSP1 [10 µM], ±Fer-1 [1 µM] for another 24 h. DRAQ7 [100 nM] was added to all wells to visualize dead cells. Images were acquired at ×10 magnification every 2 h using the IncuCyte S3 bioimaging platform. **e** Levels of NRF2, FSP1, GCLC and HO-1 cDNA were quantified by qPCR in Rasless MEFs expressing KRAS WT after cells were treated for 24 h with TBHQ [25 nM]. Fold change relative to controls is shown. **f** log2 mRNA expression data of A549 cells transfected with either siRNAs targeting NRF2 or GFP [33]. NRF2 mRNA expression is shown. **g** data as in f were analyzed for FSP1 (AIFM2) mRNA expression. Data are means ± SEM of three independent experiments in each individual cell line or representative images were applicable. Two-tailed *t*-test (**b, c, e, f, g**), Two-way ANOVA + Tukey’s multiple comparison test (**d**), ****p < 0.0001, ***p < 0.001, **p < 0.01, *p < 0.05. Uncropped blots are provided as Original Data file.

To thoroughly test a direct mechanistic link of oncogenic KRAS and FSP1 induction, we generated primary MEFs with inducible expression of KRAS^G12D^ from its endogenous locus similar to an established approach [31] (LsL-KRAS^G12D^-inducible MEFs). As expected, induction of KRAS^G12D^ by 4-hydroxytamoxifen (4OHT) treatment led to enhanced basal phosphorylation of ERK which was further increased upon refeeding with FCS (Fig. 4a). Moreover, KRAS^G12D^ induction readily elevated expression of the established MAPK target gene dual specificity phosphatase 6 (DUSP6) along with the antioxidant transcription factor nuclear factor erythroid 2-related factor 2 (NRF2) and its bona fide target genes glutamate-cysteine ligase catalytic subunit (GCLC) and heme oxigenase-1 (HO-1) (Fig. 4b). Strikingly, induction of KRAS^G12D^ was indeed sufficient to also directly induce FSP1 expression (Fig. 4b). In order to validate direct induction of FSP1 by oncogenic RAS, we validated this finding in human pancreatic duct epithelial cells (HPDE) with doxycycline-inducible expression of KRAS^G12D^ and HRAS^G12V^-inducible NIH-3T3 cells (Supplementary Fig. 4a, b). Given that oncogenic KRAS is known to induce NRF2 [31] and FSP1 was very recently shown to be a direct transcriptional target of NRF2 [32] in NSCLC mutated in the NRF2 inhibitor kelch-like ECH-associated protein 1 (KEAP1), we next tested whether activating endogenous NRF2 would be sufficient in our cells to induce FSP1. Strikingly, silencing of KEAP1 readily induced FSP1 along with NRF2 target genes (Fig. 4c). Moreover, KEAP1 knockdown rendered KRAS WT cells resistant to ferroptosis, which could partially be reverted by iFSP1 treatment (Fig. 4d).

Furthermore, treatment with the chemical NRF2 activator tert-butylhydrochinone (TBHQ) equally induced NRF2 target genes along with FSP1 (Fig. 4e). While silencing of NRF2 was toxic to MEFs (a toxicity blockable by Fer-1), we mined publicly available datasets from the KRAS-mutated NSCLC cell line A549 in which NRF2 was knocked down (Fig. 4f) [33]. Indeed, upon NRF2 silencing, FSP1 expression was also significantly decreased (Fig. 4g) confirming NRF2-mediated FSP1 regulation to be present in KRAS-mutated cells. These data suggest that oncogenic KRAS-induced NRF2 directly leads to elevated transcription of FSP1 thereby protecting them from ferroptosis. To also determine which other major KRAS effector pathway may upregulate FSP1, KRAS-mutated cells were treated with the MEK inhibitor PD184352 to block the MAPK arm downstream of KRAS as well as the AKT inhibitor MK2206. Interestingly, FSP1 mRNA was reduced only under MEK but not AKT inhibition indicating that MAPK pathway activation is responsible for elevated FSP1 levels in KRAS-mutated cells (Supplementary Fig. 4c). Of note, MEK inhibition in WT cells did not significantly regulate FSP1 mRNA despite effectively blunting expression of the established MAPK pathway target gene DUSP6 indicating preferential regulation in KRAS-mutated cells (Supplementary Fig. 4d). In support of this, MEK inhibition resulted in dose-dependent reduction in FSP1 protein levels along with decreased phosphorylation of ERK (Supplementary Fig. 4e). Furthermore, gene-set enrichment analysis of genes co-expressed with FSP1 in the lung adenocarcinoma (LUAD) TCGA dataset were significantly enriched for the MAPK pathway as indicated by enrichment of the RAF and MEK pathways (Supplementary Fig. 4f). Of note, NRF2 has been reported to be phosphorylated and activated by MAPK signaling [34]. Therefore, we also measured expression of the NRF2 target gene GCLC under MEK as compared to AKT inhibition. Indeed, GCLC was reduced by inhibition of MEK but not AKT (Supplementary Fig. 4g). Taken together, our data propose that FSP1 is upregulated in KRAS-mutated cells as a direct result of MAPK and NRF2 pathway activation.

### FSP1 aids KRAS-mediated cellular transformation and promotes tumor onset in vivo

**Fig. 5:**
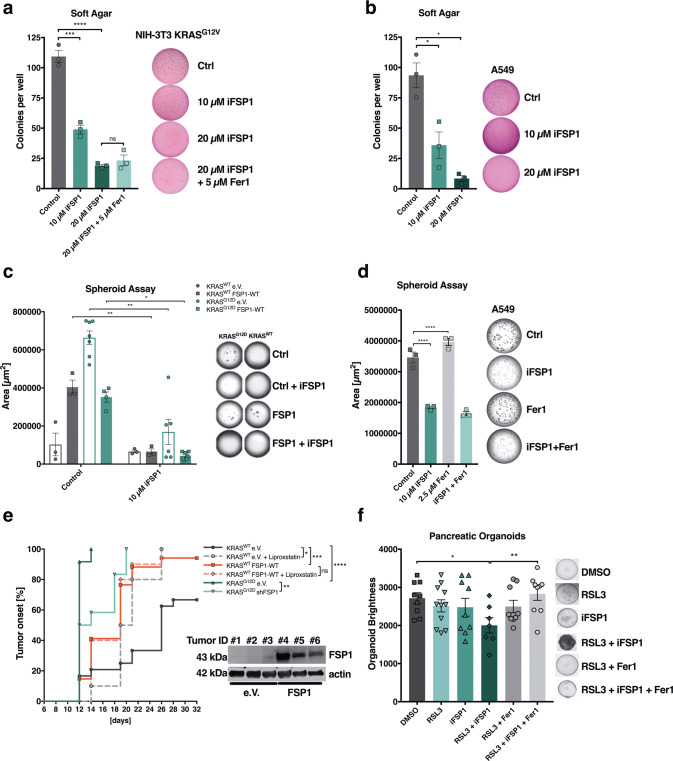
FSP1 aids cellular transformation and promotes tumor onset in vivo. **a** NIH-3T3 KRAS^G12V^ cells were treated either with DMSO, iFSP1 [10 µM, 20 µM], Fer-1 [5 µM] or both and subjected to soft agar assays for 18 days. Colony images were quantified using ImageJ. **b** The human NSCLC cell line A549 was treated as indicated and subjected to growth in soft agar for 30 days. Image analysis was done as in (**a**). **c** Indicated cells were grown in Matrigel for spheroid formation under the indicated treatment for 14 days. Images were quantified using the BZ-H4M/Measurement Application Software (Keyence). **d** A549 cells were subjected to spheroid assay growth for 9 days and treated either with DMSO, iFSP1 [10 µM], Fer-1 [2,5 µM] or both. Images were quantified using the BZ-H4M/Measurement Application Software (Keyence). **c** 8-weeks old male nude mice were injected with 5 × 10^5^ cells of the indicated cell lines (G12D e.V. (empty Vector) *n* = 11; G12D shFSP1 *n* = 12; WT e.V. *n* = 24 + Vehicle; WT e.V. + Liproxstatin-1 n = 10; WT FSP1 *n* = 24 + Vehicle; WT FSP1 + Liproxstatin-1 *n* = 10) into both flanks. Mice were injected 5× per week either with vehicle (PBS with 1% DMSO) or Liproxstatin-1 (10 mg/kg). Time until palpable tumors (min. 2 × 2 mm) were detected is depicted (tumor onset). Representative ex vivo tumors were analyzed for FSP1 expression. **f** Pancreatic organoids were treated with DMSO, RSL3 [100 nM] or iFSP1 [10 µM] alone or in combination with Ferrostatin-1 (Fer-1) [5 µM] for 48 h. Images were quantified using the BZ-H4M/Measurement Application Software (Keyence). Data are means ± SEM of at least three independent experiments in each individual cell line or representative images were applicable. Two-way ANOVA (**a**, **b**, **c**, **d**), log-rank test (**e**), two-tailed *t*-test (**f**), ****p < 0.0001, ***p < 0.001, **p < 0.01, *p < 0.05.

One hallmark of oncogenic KRAS is its capacity to mediate cellular transformation. Therefore, we hypothesized that FSP1 activity may aid cellular transformation capacity of KRAS-mutated cells. Indeed, 3T3 cells transformed by KRAS^G12V^ expression presented with decreased colony formation in soft agar in the presence of iFSP1. Yet, this activity of FSP1 was not due to ferroptosis protection as co-treatment with Fer-1 did not rescue decreased colony formation (Fig. 5a). Moreover, human KRAS-mutated A549 cells equally showed decreased soft agar colony formation in the presence of iFSP1 (Fig. 5b) suggesting that elevated FSP1 expression additionally may promote cellular transformation of KRAS-mutated cells independently of ferroptosis protection. Recently, in 3D Matrigel-based spheroid assays, ferroptosis was shown to occur in spheroid centers thereby limiting their growth [35]. Therefore, we next tested the extent of spheroid formation in KRAS-mutated as compared to WT cells in this experimental system. As expected, KRAS-mutated cells formed spheroids much more efficiently than KRAS WT cells although a few colonies could be detected likely due to some extent of spontaneous transformation enabling continuous proliferation of KRAS WT MEFs (Fig. 5c). Here, overexpression of FSP1 was sufficient to allow for spheroid growth in KRAS WT cells to a similar extent as KRAS-mutant cells. While FSP1 overexpression in KRAS-mutant cells led to a slight decrease in colony formation, importantly, iFSP1 incubation readily reverted spheroid formation enabled by FSP1 overexpression in both cases (Fig. 5c). Moreover, iFSP1 also significantly impacted spheroid formation of human A549 cells yet again, this activity was not caused by protecting from ferroptosis (Fig. 5d, Supplementary Fig. 5a). Thereby, our data support the concept that cellular transformation endowed by KRAS may at least in part depend upon FSP1 expression and activity, yet this activity is independent of its role in ferroptosis protection. Next, we aimed to test whether FSP1 expression was sufficient to impact tumor initiation. To this end, we transplanted mice with KRAS WT cells with either control or FSP1 overexpression in comparison to KRAS-mutant control and cells with short hairpin RNA (shRNA)-mediated FSP1 silencing (Supplementary Fig. 5b). As expected, KRAS-mutated cells presented with earlier tumor onset than KRAS WT cells. Yet strikingly, FSP1 expression was sufficient in KRAS WT cells to significantly accelerate tumor onset and increase tumor incidence closer to the rates of KRAS-mutant tumors (Fig. 5e). Moreover, treatment of mice bearing KRAS WT tumors with the ferroptosis-selective inhibitor Liproxstatin-1 accelerated tumor onset to similar levels as FSP1 overexpression suggesting ferroptosis to be indeed responsible for suppression of tumor initiation capacity of WT cells in vivo. Importantly, Liproxstatin-1 treatment of FSP1-overexpressing WT tumors did not result in additional promotion of tumor onset indicating FSP1 to promote KRAS WT tumor initiation by protecting from ferroptosis. Interestingly, and, similar to our findings in spheroid assays, tumor volumes of tumors arising also increased when expressing exogenous FSP1, yet this increase was independent of ferroptosis protection as Liproxstatin-1 treatment did not affect tumor volumes (Supplementary Fig. 5c). Vice versa, FSP1 silencing in KRAS-mutated tumors delayed tumor onset albeit not to the levels of KRAS WT tumors. Therefore, oncogenic KRAS partially promotes its early tumor onset through FSP1-mediated ferroptosis protection. Moreover, FSP1 expression alone is sufficient to promote tumor initiation in the absence of oncogenic KRAS by suppressing ferroptosis in vivo.

Oncogenic mutations in KRAS are most frequently observed in patients with pancreatic ductal adenocarcinoma (PDAC). Yet, most small molecules inducing ferroptosis do not display pharmacokinetics and solubilities suitable for in vivo use yet. Therefore, we next generated organoids from mice developing pancreatic intraepithelial neoplasia (PanINs) as a result of KRAS^G12D^ expression from the endogenous promotor [36] and treated them either with RSL3 alone or in combination with iFSP1. Strikingly, RSL3 treatment alone was insufficient to induce ferroptosis in pancreatic organoids but the combination with iFSP1 led to effective killing of pancreatic organoids expressing KRAS^G12D^ (Fig. 5f). Based on these data, we propose that breaking ferroptosis resistance through the use of FSP1 inhibitors might be a particularly potent treatment strategy against KRAS-driven cancers.

### FSP1 expression is upregulated in KRAS-mutated cancers and correlates with poor outcome in PDAC patients

**Fig. 6:**
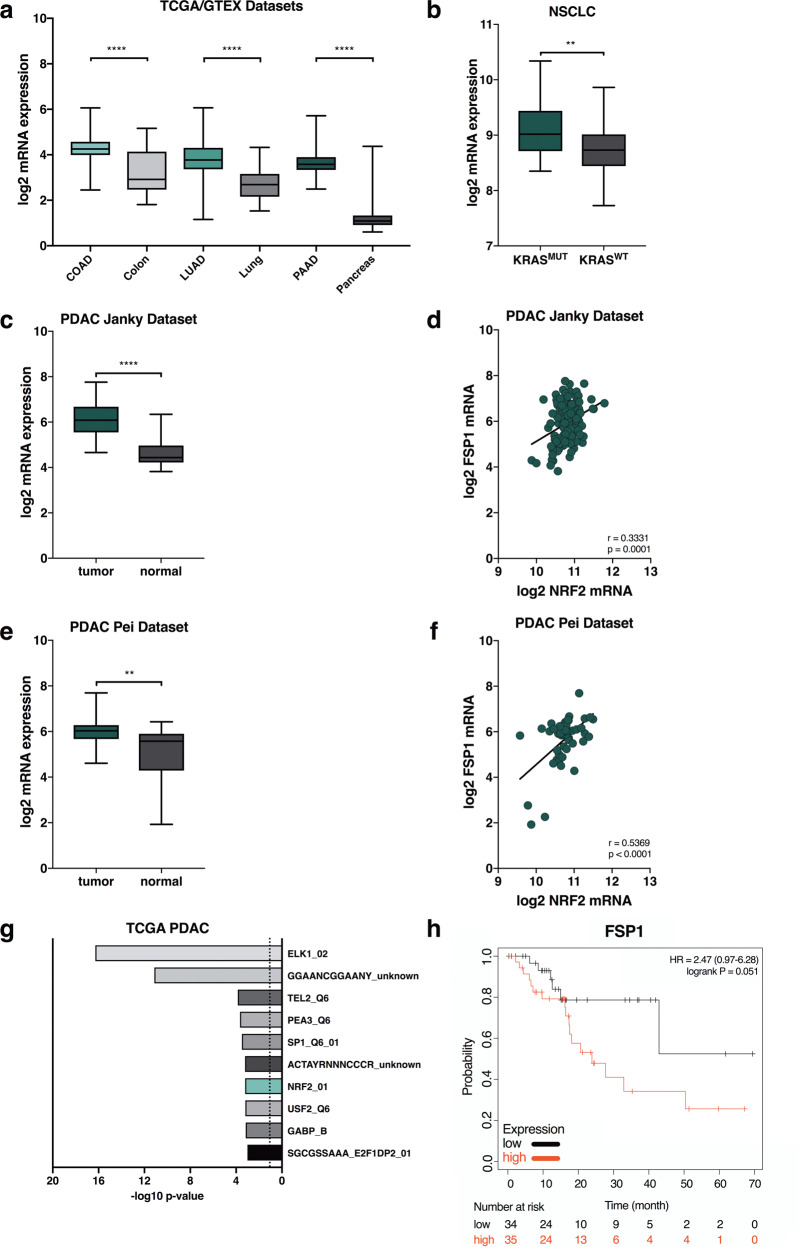
FSP1 is upregulated in KRAS-mutated cancers and correlates with poor relapse-free survival. **a** Log^2^-transformed RPKM expression data for FSP1 (AIFM2) for the indicated TCGA tumor (COAD-colon adenocarcinoma; LUAD-lung adenocarcinoma; PAAD-pancreatic adenocarcinoma) or GTEX normal control datasets are plotted. **b** NSCLC expression data (GSE31852) were split into two groups by KRAS-mutation status (KRAS-mutated *n* = 24, KRAS WT *n* = 100) and analyzed for log^2^ FSP1 expression. **c, d** PDAC expression data from Janky et al. [38] tumor n = 118, normal n = 13 were analyzed for log2 FSP1 expression in tumor as compared to adjacent normal as well as co-expression of FSP1 with NRF2 within tumor tissue. **e, f** PDAC expression data from Pei et al. [37] tumor *n* = 36, normal *n* = 15 were analyzed as in **c, d**. **g** Genes significantly co-expressed with FSP1 (*r* ≥ 0.3, FDR < 0.01) within the PDAC TCGA dataset were analyzed for transcription factor binding motifs by gene-set enrichment (GSEA) and -log10-transformed false discovery rates (FDR) of significantly enriched motifs are plotted. **h** Kaplan Meier survival of relapse-free survival in PDAC patients *n* = 69 according to FSP1 expression high versus low split by median is shown. Data were analyzed by and downloaded from KM plotter [58]. Whiskers are shown from min to max. Two-tailed *t* test (**a, b, c, e**) and log-rank test (**h**), ****p < 0.0001, **p < 0.01.

In order to test whether FSP1 expression may be upregulated in KRAS-driven cancer, we probed publicly available tumor (TCGA) and normal (GTEX) datasets for FSP1 expression in colorectal cancer, lung adenocarcinoma and pancreatic adenocarcinoma in comparison to their respective normal tissue of origin using Gene Expression Profiling Interactive Analysis (GEPIA). Interestingly, FSP1 was significantly overexpressed in all three tumor types as compared to the respective normal tissues (Fig. 6a). While in pancreatic cancer the vast majority of patients present with activating KRAS mutations and a KRAS WT group is therefore difficult to obtain, in non-small cell lung cancer (NSCLC) about half of the patients are usually WT. Strikingly, when dividing an NSCLC dataset (GSE31852) by KRAS-mutation status, FSP1 expression was significantly upregulated in KRAS-mutated patient material (Fig. 6b). Moreover, in two independent PDAC datasets [37, 38] with included adjacent normal tissue, FSP1 expression was significantly upregulated in PDAC over normal pancreas and FSP1 mRNA correlated with NRF2 mRNA within these two datasets (Fig. 6c–f). Moreover, we performed gene-set enrichment analysis (GSEA) on transcription factor motifs of genes co-expressed with FSP1 within the TCGA PDAC dataset. Strikingly, NRF2 was amongst the top 10 enriched motifs (Fig. 6g) suggesting FSP1 upregulation in PDAC patients to be a result of NRF2-mediated transcription. Lastly, high FSP1 expression in PDAC patients showed a strong trend towards drastically shortened relapse-free survival (Fig. 6h).

Taken together, our data establish that endogenous levels of oncogenic KRAS expression render cells more resistant to ferroptosis by upregulating FSP1 through the NRF2 and MAPK pathway allowing for a superior capacity to buffer acute lipid peroxidation during tumor initiation. Hence, only combined targeting of GPX4 and FSP1 is effective at killing KRAS-driven pancreatic organoids and FSP1 is upregulated in human KRAS-driven cancers. Based on these data, we propose that pro-ferroptotic therapy for KRAS-driven cancers should include inhibition of FSP1 in order to achieve efficient tumor cell killing.

## Discussion

In the present study, we identify that expression of near-endogenous levels of oncogenic KRAS renders cells more resistant to ferroptosis through elevated expression of FSP1. Of note, in earlier studies overexpression of oncogenic HRAS in fibroblasts as compared to cells expressing empty vector led to a sensitization to erastin-induced cell death [13, 39], later found to be ferroptotic due to erastin-mediated targeting of xCT [10]. What might seem as a discrepancy might in fact represent two distinct stages of cellular transformation. Upon acute overexpression of an oncogenic RAS variant, cellular levels of ROS are known to be upregulated due to NOX1 induction [6]. Moreover, in line with an earlier study by Yang et al. [40] we observed that a cluster of genes involved in iron uptake (TFRC, STEAP3) was in fact upregulated in KRAS-mutated cells (Fig. 3b), possibly to feed an increased requirement for iron in the mitochondrial respiratory chain. This may suggest that RAS expression would fuel lipid peroxidation due to elevated basal ROS which is also what was observed by Yang et al. 2008. Yet, chronic elevated levels of ROS are known to activate NRF2. In fact, endogenous expression levels as opposed to overexpression of oncogenic KRAS were shown to effectively induce NRF2 activation [31]. Along these lines, a recent study found that NRF1 and NRF2 protect cells from ferroptosis through distinct and independent mechanisms [41]. Moreover, NRF2 was recently shown to induce FSP1 transcription via NRF2 in KEAP1-mutant NSCLC [32]. In keeping with these results, we now find that cells expressing oncogenic KRAS directly induce FSP1 expression through MAPK-NRF2 pathway activation. Of note, class I FINs, which lead to GSH depletion, did not show a significant difference between KRAS-mutated and -WT cells. This observation may suggest that GSH depletion triggers general ROS accumulation which in turn activates NRF2 and via this route may upregulate FSP1 also in WT cells thereby neutralizing the difference in killing. Hence, we propose a model wherein effective activation of NRF2-mediated transcription might be decisive for whether oncogenic KRAS expression renders cells more or less sensitive to ferroptosis.

An interesting possibility is that the Nonsense-Mediated Decay (NMD) pathway might contribute to the regulation of FSP1 mRNA. NMD can regulate a number of perfectly functional transcripts many of which usually have an abnormally long 3′UTR. Indeed, FSP1 murine transcript variants contain several NMD-inducing features including an abnormally long 3′UTR in one of them. Interestingly, FSP1 mRNA was found to be upregulated in murine embryonic stem cells upon NMD inhibition [42], and oncogenic KRAS might inhibit NMD via several non-exclusive mechanisms. For instance, KRAS^G12D^ is known to activate p38 MAPK [43–45] and p38 MAPK has been shown to inhibit NMD [46]. Additionally, ER stress is well-known to inhibit NMD [47] and has been observed in KRAS^G12D^-expressing cells [48]. It is therefore tempting to speculate whether cells expressing oncogenic KRAS may inhibit NMD which in turn could result in FSP1 mRNA stabilization.

Expression of oncogenic KRAS creates selective metabolic addiction to nucleotide synthesis via the pentose phosphate pathway (PPP) [49]. Interestingly, high cellular levels of NADPH, the product of the PPP, were identified as markers of resistance to ferroptosis [50]. NADPH in turn is an important electron donor for a variety of cellular enzymes including FSP1. Consequently, high-level exogenous overexpression of FSP1, as obtained in our experiments, may impact cellular NADPH levels and, with that, be problematic for metabolic NADPH addiction of KRAS-mutant cells [49]. In line with this, FSP1 overexpression in KRAS-mutant cells decreased their capacity to form spheroids. Moreover, inhibition of the PPP was shown to reduce soft agar colony formation of transformed 3T3 MEFs [51]. In favor of another ferroptosis-independent function for FSP1 expression in cancer, we found that tumor volume growth in WT cells in vivo was promoted by FSP1 expression but not Liproxstatin-1 treatment. This growth promotion may be facilitated by FSP1- generated NAD+ which was shown to promote glycolysis [52], a mode of energy generation advantageous for hypoxic tumors.

Interestingly, two recent studies identified GTP cyclohydrolase-1 (GCH1) and its products tetrahydrobiopterin /dihydrobiopterin (BH_4/_BH_2_) to act as potent cellular antioxidants protecting from ferroptosis in the absence of GPX4 [53, 54]. Yet, cells not expressing GCH1 seem to solely depend upon FSP1 for the generation of endogenous radical-trapping agents, a fact used for screening for novel FSP1 inhibitors [55]. Of note, GCH1 mRNA expression was barely detectable in our cellular systems and also not influenced by KRAS-mutation status in contrast to FSP1 (data not shown).

In a genetically engineered mouse model of KRAS-driven PDAC, inducible whole-body deletion of xCT led to significant tumor regression [56]. However interestingly, cancer-associated fibroblast (CAF)-restricted deletion of xCT in the very same mouse model was sufficient to achieve a strong anti-tumor effect [57]. These data together with the fact that GPX4 deletion within PanINs was insufficient to trigger ferroptosis in pancreatic cancer [14], support the idea that KRAS-mutated cells have evolved an additional layer of protection against ferroptotic cell death. Our data propose that elevated FSP1 expression in KRAS-mutated cells is, at least in part, responsible for this protection. Based on these considerations, we propose that combined induction of ferroptosis and FSP1 inhibition should be considered for therapeutic strategies developed against KRAS-mutated cancers.

## Supplementary information

author change agreemen 2

author change agreement 1

author change agreemen 3

Supplementary Information

author checklist

## Data Availability

The data and material that support the findings of this study are available from the corresponding author upon reasonable request.
