# Supplementary material for: Elevated FSP1 protects KRAS-mutated cells from ferroptosis during tumor initiation
Source: Cell Death Differ. 2022 Nov 29;30(2):442–56. doi: 10.1038/s41418-022-01096-8 (PMC9950476; doi:10.1038/s41418-022-01096-8)
Supplement: Supplementary file 1 — author change agreemen 2 [file 41418_2022_1096_MOESM1_ESM.docx]

I agree that Julia Beck and Keiko Nakayama will be included.

________________________

Fabienne Müller, M.Sc.
AG Silvia von Karstedt

Department of Translational Genomics/

CECAD
Joseph-Stelzmann-Str. 26
50931 Cologne

fabienne.mueller@uk-koeln.de

Hi Silvia,

I am happy with the author changes/additions.

Best,

Jonathan

Dear Silvia, 

I hereby agree to the inclusion of the two additional authors Julia Beck and Keiko Nakayama. 

Best
Christina

Hello Silvia,

I agree with adding Julia and Keiko to the list of authors.

Best

Eric

Dear Silvia,

I am happy for Julia Beck and Keiko Nakayama to be included into the revised version of the manuscript.

Best wishes,
Sofya Tishina

Quoting Silvia von Karstedt <[svonkars@uni-koeln.de](mailto:svonkars@uni-koeln.de)>:

Dear co-authors,

for the submission to Cell Death and Differentiation to proceed, we will need a written agreement via  e-mal to me from everyone that you are happy for Julia Beck and Keiko Nakayama to be included into the revised version of the manuscript. Please send this to me ASAP.

all the best,

Silvia

-- 
Sofya Tishina
Department of Translational Genomics/
CECAD
AG von Karstedt
Joseph-Stelzmann-Str. 26
D-50931 Cologne

email: [stishina@uni-koeln.de](mailto:stishina@uni-koeln.de)

Hello,

I agree with the addition of the two new authors.

 Best wishes

Jenny Stroh

Hi Silvia,

I agree, this is fine by me

Best,

Quoting Silvia von Karstedt <[svonkars@uni-koeln.de](mailto:svonkars@uni-koeln.de)>:

Dear co-authors,

for the submission to Cell Death and Differentiation to proceed, we will need a written agreement via  e-mal to me from everyone that you are happy for Julia Beck and Keiko Nakayama to be included into the revised version of the manuscript. Please send this to me ASAP.

all the best,

Silvia

-- 
Fatma Isil Yapici, M.Sc.
AG von Karstedt
Department of Translational Genomics/
CECAD
Joseph-Stelzmann-Str. 26
50931 Cologne

Tel.: +49 (0)221 / 478 84327
Email: [fyapici@uni-koeln.de](mailto:fyapici@uni-koeln.de)

Dear Silvia,

Hereby I express my agreement with the new authors inclusion. Good luck with the resubmission!

Kind regards,

Lucia.

Dear Silvia,

I agree to the new authorship order and the inclusion of Julia Beck and Keiko Nakayama as co-authors.

Best,

Johannes

Dear Silvia,

I agree and I am fine that Julia Beck and Keiko Nakayama are now added as authors on Fabienne's manuscript CDD-22-0834R.

Best,

Gabriel

Gabriel LEPRIVIER, PhD

Group Leader, AG "Stress responses and Cancer"

Institute of Neuropathology

University Hospital Düsseldorf

Moorenstr. 5

40225 Düsseldorf

Phone: +49 (0)211 81 08748

Email: Gabriel.Leprivier@med.uni-duesseldorf.de

I agree that Julia Beck and Keiko Nakayama will be included. 

Best, 
Alina Dahlhaus

Alina Dahlhaus

Department of Translational Genomics/
CECAD
AG von Karstedt
Joseph-Stelzmann-Str. 26
D-50931 Cologne

Tel.: +49 (0)221 / 478 84327
email: adahlha1@uni-koeln.de/

alina.dahlhaus@uk-koeln.de
