# Supplementary material for: Elevated FSP1 protects KRAS-mutated cells from ferroptosis during tumor initiation
Source: Cell Death Differ. 2022 Nov 29;30(2):442–56. doi: 10.1038/s41418-022-01096-8 (PMC9950476; doi:10.1038/s41418-022-01096-8)
Supplement: Supplementary file 2 — author change agreement 1 [file 41418_2022_1096_MOESM2_ESM.pdf]

SPRINGER NATURE

Change of authorship request form - Journals (pre-acceptance)

	First name	Family name		Signature	Date
8th author	Julia	Beck	~~I agree to the proposed new authorship shown in section 4~~ /**and the addition/removal*of my name to the authorship list** ~~/and the proposed change in corresponding author~~		
9th author			I agree to the proposed new authorship shown in section 4 /**and the addition/removal*of my name to the authorship list** /and the proposed change in corresponding author		
10th author	Keiko	Nakayama	~~I agree to the proposed new authorship shown in section 4~~ /**and the addition/removal*of my name to the authorship list** ~~/and the proposed change in corresponding author~~	Keiko Nakayama	Oct. 7, 2022

Please use an additional sheet if there are more than 10 authors.

In case of author collaborations with formal agreement:

	Name of consortium/consortia	First name	Family name		Signature	Date
Representative/legal guarantor				I agree to the proposed new authorship shown in section 4 /**and the addition/removal*of my name to the authorship list** /and the proposed change in corresponding author		

Both added/removed authors should complete the information in the first table under Section 6.

---- End of form ----

SPRINGER NATURE Springer Nature is one of the world's leading global research, educational and professional publishers, created in May 2015 through the combination of Nature Publishing Group, Palgrave Macmillan, Macmillan Education and Springer Science+Business Media.
