# Supplementary material for: Elevated FSP1 protects KRAS-mutated cells from ferroptosis during tumor initiation
Source: Cell Death Differ. 2022 Nov 29;30(2):442–56. doi: 10.1038/s41418-022-01096-8 (PMC9950476; doi:10.1038/s41418-022-01096-8)
Supplement: Supplementary file 4 — Supplementary Information [file 41418_2022_1096_MOESM4_ESM.docx]

**Supplementary Information**

**Elevated FSP1 protects KRAS-mutated cells from ferroptosis during tumor initiation**

**Fabienne Müller^1, 2^, Jonathan K. M. Lim^3^, Christina M. Bebber^1, 2^, Eric Seidel^1, 2^, Sofya Tishina^1, 2^, Alina Dahlhaus^1, 2^, Jenny Stroh^1, 2^, Julia Beck^1, 2^, Fatma Isil Yapici^1, 2^, Keiko Nakayama^4^, Lucia Torres Fernández^1^, Johannes Brägelmann^1, 5, 6^, Gabriel Leprivier^3^ & Silvia von Karstedt ^1, 2, 5, #^**

^1^University of Cologne, Faculty of Medicine and University Hospital Cologne, Department of Translational Genomics, Cologne, Germany.

^2^CECAD Cluster of Excellence, University of Cologne, Cologne, Germany.

^3^Heinrich Heine University, Medical Faculty and University Hospital Düsseldorf, Institute of Neuropathology, Düsseldorf, Germany.

^4^Division of Cell Proliferation, ART, Graduate School of Medicine, Tohoku University, Sendai, Japan

^5^University of Cologne, Faculty of Medicine and University Hospital Cologne, Center for Molecular Medicine Cologne, Cologne, Germany.

^6^Mildred Scheel School of Oncology Cologne, Faculty of Medicine and University Hospital Cologne, University of Cologne, Cologne, Germany.

^#^Corresponding author: S von Karstedt, University of Cologne, Faculty of Medicine and University Hospital Cologne, Department of Translational Genomics, Cologne, Germany. CECAD Research Building, Joseph-Stelzmann-Str. 26, 50931 Cologne, Germany. Tel.: +49 (0)221 / 478 84340; E-mail: [s.vonkarstedt@uni-koeln.de](mailto:s.vonkarstedt@uni-koeln.de)

** Supplementary Fig. 1 Oncogenic KRAS activity renders cells resistant to ferroptosis.** **a** KRAS WT or KRAS^G12D^-expressing MEFs were treated with either erastin [0.37 µM], SAS [ 0.17 mM], IKE [1.11 µM], RSL3 [120 nM], ML210 [0.37 µM] or ML162 [1.11 µM] for 24 h. DRAQ7 [100 nM] was added to visualize dead cells. Dead cells were analyzed by using the IncuCyte quantification software. Images were acquired at 10x magnification every 2 h using the IncuCyte S3 bioimaging platform. **b, c** KRAS WT or KRAS^G12D^-expressing MEFs were treated with or without RSL3 [100 nM] for 48 h and confluence was determined by analyzing phase contrast using the IncuCyte quantification software or DRAQ7 [100 nM] (**c**) was added to wells to visualize dead cells. Images were acquired at 10x magnification every 4 h using the IncuCyte S3 bioimaging platform. **d** KRAS WT or KRAS^G12C^-expressing MEFs were treated with RSL3 [50 nM], AMG510 [500 nM] and Fer1 [2.5 µM] alone or in combination for 24 h. Cell viability was determined by Cell Titer blue. % viability is calculated relative to untreated KRAS WT MEFs (100%). **e** 3LL cells were treated with RSL3 [100 nM], AMG510 [10 µM], ARS1620 [10 µM] and Fer1 [5 µM] alone or in combination for 24 h. Cell death was determined by propidium iodide (PI) uptake and flow cytometry. Gates are set on PI+ cells in control untreated cells. Data are means +/- SEM of three independent experiments in each individual cell line. Two-way ANOVA + Tukey’s multiple comparison test (**a,** **d, e**), two-tailed t-test of end timepoint (**c**), **** p<0.0001, ** p<0.01, * p<0.05.

**Supplementary Fig. 2 Comparative lipidomics in KRAS WT and KRAS^G12D^-expressing cells.** **a** Bulk-sorted MEFs expressing KRAS^WT^ or KRAS^G12D^ were treated either with DMSO, RSL3 [100 nM] alone or in combination with Ferrostatin-1 (Fer-1) [5 µM] for 5 h and stained for lipid ROS accumulation using BODIPY C11. Cells were analyzed by flow cytometry. Negative gates were placed based on DMSO controls. **b - e** KRAS WT expressing cells (n = 5 samples) as compared to KRAS^G12D^- expressing cells (n = 5 samples) were analyzed for basal diacylglycerol (DAG) and ether-linked lipids by mass spectrometry. Lipid content was normalized to infused protein for each condition and replicate. Individual PUFAs (4 double bonds or more) are plotted. **f** Data showing the representation of mono-oxidized phospholipid species (PE phosphatidylethanolamine; PC phosphatidylcholine) in KRAS wild type as compared to KRAS^G12D^- expressing cells treated with either DMSO or RSL3 [100 nM] for 5 h and then subjected to lipidomics. Samples for each condition (n=5) were averaged and normalized to the cell number (2.5x10^6^). nmoles lipid/mg protein lysate is shown. **g** Rasless MEFs expressing KRAS wild type or KRAS^G12D^ were treated either with DMSO, RSL3 [100 nM] alone or in combination with Ferrostatin-1 (Fer-1) [5 µM] for 5 h and stained for ROS accumulation using H2DCFDA. Cells were analyzed by flow cytometry. Negative gates were placed based on DMSO controls. Data are means +/- SEM of three independent experiments in each individual cell line. Two-way ANOVA + Tukey’s multiple comparison test, *** p<0.001, ** p<0.01, * p<0.05.

**Supplementary Fig. 3 RNA expression of ferroptosis regulators.** **a** RNA-seq expression data in FPKM (fragments per kilobase of exon model per million reads mapped) from KRAS WT and KRAS^G12D^- expressing cells were log2 transformed (+0.01) and plotted for relative expression of genes involved in ferroptosis. **b** Ratio of total oxidized and reduced nicotinamide adenine dinucleotide phosphates (NADP+ and NADPH, respectively) was measured in lysates from 20,000 cells of either KRAS WT or KRAS^G12D^- expressing cells using the NADP/NADPH-Glo™ kit (Promega). **c** KRAS WT or KRAS^G12D^-expressing cells were treated with RSL3 [100 nM] alone or in combination with rising concentrations of iFSP1. Cell viability was determined by Cell Titer blue. % viability is calculated relative to untreated KRAS WT MEFs (100%). **d** Cell death of cells treated as in (**c**) was determined by flow cytometry and propidium iodide (PI) incorporation. Gates are set on PI+ cells in control untreated cells. Data are means +/- SEM of three independent experiments in each individual cell line. Two-tailed t-tests (**a**), Two-way ANOVA + Tukey’s multiple comparison test (**c, d**), **** p<0.0001, * p<0.05.

**Supplementary Fig. 4 MEK activity elevates FSP1 mRNA levels.** **a** Levels of the indicated cDNAs were quantified by qPCR in KRAS^G12D^-inducible HPDE cells after 72 h of doxycycline [0.5 µg/ml] treatment. Fold change relative to controls is shown. **b** Levels of the indicated cDNAs were quantified by qPCR in HRAS^G12V^-inducible NIH-3T3 cells after 48 h of tamoxifen (4OHT) treatment [100 nM]. Fold change relative to controls is shown. **c** Levels of FSP1 cDNA were quantified by qPCR in Rasless MEFs expressing WT or KRAS^G12D^ treated with either DMSO, iMEK (PD184352) [5 µM] or iAKT (MK2206) [5 µM] for 48 h. Fold change relative to DMSO KRAS WT controls is shown. **d** Levels of DUSP6 cDNA were determined in cells treated as in (**c**). **e** KRAS^G12D^-expressing cells were treated with the indicated concentrations of iMEK (PD184352) for 72 h, lyzed and subjected to protein analysis by Western blotting. **f** Genes significantly co-expressed with FSP1 within the LUAD TCGA dataset were analyzed by gene-set enrichment (GSEA) and -log^10^-transformed false discovery rates (FDR) of significantly enriched gene sets are plotted. **g** Levels of GCLC cDNA were determined in cells treated as in (**c**). Data are means +/- SEM of at least two independent experiments in each individual cell line. Two-tailed t-test, *** p<0.001, ** p<0.01, * p<0.05.

**Supplementary Fig. 5 Spheroid growth and FSP1 silencing controls. a** A549 cells were subjected to spheroid assay growth for 4 days and treated as indicated. Images were quantified using the BZ-H4M/Measurement Application Software (Keyence). **b** Levels of FSP1 cDNA were quantified by qPCR in Rasless MEFs expressing either KRAS^G12D^ pLKO.1 empty Vector or KRAS^G12D^ pLKO.1 shFSP1 stable expression plasmids. **c** 8-weeks old male nude mice were injected with 5 x 10^5^ cells of the indicated cell lines (G12D e.V. (empty Vector) n=11; G12D shFSP1 n=12; WT e.V. n=24 + Vehicle; WT e.V. + Liproxstatin-1 n=10; WT FSP1 n=24 + Vehicle; WT FSP1 + Liproxstatin-1 n=10) into both flanks. Mice were injected 5x per week either with vehicle [PBS with 1% DMSO] or Liproxstatin-1 [10 mg/kg]. Tumor length and width were measured by caliper at day 26 and volume was calculated as (length x width x width)/2. Data are means +/- SEM of three independent experiments in each individual cell line. Two-way ANOVA + Tukey’s multiple comparison test (**a,** **c**), two-tailed t-test (**b**), **** p<0.0001, *** p<0.001.

**Supplementary Table 1 qPCR primers**

| **Primer mouse** | **Sequence** |
| --- | --- |
| mouse FSP1 FWD | TGCCTCGCAATGAGTATCGG |
| mouse FSP1 REV | GCCAGCCTACTCTCTGCAAAT |
| mouse DUSP6 FWD | ATAGATACGCTCAGACCCGTG |
| mouse DUSP6 REV | ATCAGCAGAAGCCGTTCGTT |
| mouse NRF2 FWD | GCTGCTCGGACTAGCCATTG |
| mouse NRF2 REV | TCAAATCCATGTCCTGCTGGG |
| mouse GCLC FWD | GGACAAACCCCAACCATCC |
| mouse GCLC REV | GTTGAACTCAGACATCGTTCCT |
| mouse HO-1 FWD | GCCGAGAATGCTGAGTTCATG |
| mouse HO-1 REV | TGGTACAAGGAAGCCATCACC |
| mouse Rpl13a FWD | AGCCTACCAGAAAGTTTGCTTAC |
| mouse Rpl13a REV | GCTTCTTCTTCCGATAGTGCATC |
| mouse Rplp0 FWD | TAAAGACTGGAGACAAGGTG |
| mouse Rplp0 REV | GTGTACTCAGTCTCCACAGA |
| mouse Actin FWD | GGCTGTATTCCCCTCCATCG |
| mouse Actin REV | CCAGTTGGTAACAATGCCATGT |

| **Primer human** | **Sequence** |
| --- | --- |
| human FSP1 FWD | GACTCCTTCCACCACAATGTGG |
| human FSP1 REV | CAGCACCATCTGGTTCTTCAGG |
| human DUSP6 FWD | GCAATACTTTGGGTTGGTTTC |
| human DUSP6 REV | AACTCTCCCTTCTTCACAATC |
| human NRF2 FWD | CCAACTACTCCCAGGTTGCC |
| human NRF2 REV | AGTGACTGAAACGTAGCCGAA |
| human HO-1 FWD | ACCTTCCCCAACATTGCCAG |
| human HO-1 REV | CAACTCCTCAAAGAGCTGGATG |
| human 18S FWD | GCAGAATCCACGCCAGTACAAG |
| human 18S REV | GCTTGTTGTCCAGACCATTGGC |
